# Packing Amalgam

**Published:** 1900-04-15

**Authors:** C. P. Pruyn


Packing Amalgam.—A very good object lesson for any one is to use glass tubes for packing amalgam. Take a glass tube about the size of a lead pencil, one-quarter to one-half inch long, shellac it upon a piece of board, then wind about the glass tube some paper, and proceed to insert the filling as you ordinarily do in practice. Remove the paper, and carefully observe the air spaces that you will see between the filling and the glass tube; then take another tube without the paper wound about it, and proceed as before, carefully examining each step of the operation as you go on, and carefully note the extreme care that will be necessary to thoroughly adapt the amalgam to all the sides of the glass, so that no air spaces can be seen.

While this is a simple experiment and can be made very easily, it is an object lesson that I think every operator should witness.

While a dozen different men might fill a dozen different holes in a steel plate with the same make of amalgam, and very little difference would result in the measurement afterwards, I am convinced that when it comes to the practical work in the mouth, the personal equation forms a much larger factor than we are oftentimes led to believe.—*Dr. C. P. Pruyn, Dental Century.*

